# (*E*)-*N*′-(2-Chloro-5-nitro­benzyl­idene)-4-methoxy­benzohydrazide

**DOI:** 10.1107/S1600536808034107

**Published:** 2008-10-25

**Authors:** Hong-Yan Ban, Cong-Ming Li

**Affiliations:** a School of Chemical Engineering, University of Science and Technology Liaoning, Anshan 114051, People’s Republic of China; b College of Sciences, Shenyang University, Shenyang 110044, People’s Republic of China

## Abstract

In the title compound, C_15_H_12_ClN_3_O_4_, the benzohydrazide group is not planar and the mol­ecule exists in a *trans* configuration with respect to the methyl­idene unit. The dihedral angle between the two substituted benzene rings is 0.4 (3)°. In the crystal structure, mol­ecules are linked by inter­molecular N—H⋯O hydrogen bonds, forming chains parallel to the *c* axis.

## Related literature

For the biological activity of hydrazones, see: Zhong *et al.* (2007 ▶); Raj *et al.* (2007 ▶); Jimenez-Pulido *et al.* (2008 ▶). For related structures, see: Yehye *et al.* (2008 ▶); Fun, Patil, Jebas *et al.* (2008 ▶); Yang *et al.* (2008 ▶); Ejsmont *et al.* (2008 ▶); Fun, Patil, Rao *et al.* (2008 ▶). For reference bond lengths, see: Allen *et al.* (1987 ▶).
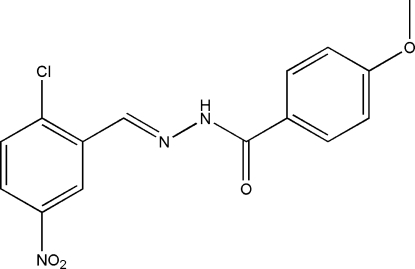


## Experimental

### 

#### Crystal data

C_15_H_12_ClN_3_O_4_
*M* *_r_* = 333.73
Monoclinic, 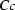

*a* = 11.724 (2) Å
*b* = 13.482 (3) Å
*c* = 9.4259 (19) Å
β = 97.199 (3)°
*V* = 1478.1 (5) Å^3^
*Z* = 4
Mo *K*α radiation
μ = 0.28 mm^−1^
*T* = 298 (2) K
0.20 × 0.20 × 0.17 mm

#### Data collection

Bruker SMART CCD area-detector diffractometer
Absorption correction: multi-scan (*SADABS*; Sheldrick, 1996 ▶) *T*_min_ = 0.946, *T*_max_ = 0.953
4284 measured reflections
2735 independent reflections
2320 reflections with *I* > 2σ(*I*)
*R*_int_ = 0.015

#### Refinement

*R*[*F* ^2^ > 2σ(*F* ^2^)] = 0.035
*wR*(*F* ^2^) = 0.089
*S* = 1.04
2735 reflections
212 parameters
3 restraints
H atoms treated by a mixture of independent and constrained refinement
Δρ_max_ = 0.14 e Å^−3^
Δρ_min_ = −0.19 e Å^−3^
Absolute structure: Flack (1983 ▶), 1079 Friedel pairs
Flack parameter: −0.01 (7)

### 

Data collection: *SMART* (Bruker, 1998 ▶); cell refinement: *SAINT* (Bruker, 1998 ▶); data reduction: *SAINT*; program(s) used to solve structure: *SHELXS97* (Sheldrick, 2008 ▶); program(s) used to refine structure: *SHELXL97* (Sheldrick, 2008 ▶); molecular graphics: *SHELXTL* (Sheldrick, 2008 ▶); software used to prepare material for publication: *SHELXTL*.

## Supplementary Material

Crystal structure: contains datablocks global, I. DOI: 10.1107/S1600536808034107/rz2255sup1.cif

Structure factors: contains datablocks I. DOI: 10.1107/S1600536808034107/rz2255Isup2.hkl

Additional supplementary materials:  crystallographic information; 3D view; checkCIF report

## Figures and Tables

**Table 1:** Hydrogen-bond geometry (Å, °)

*D*—H⋯*A*	*D*—H	H⋯*A*	*D*⋯*A*	*D*—H⋯*A*
N2—H2⋯O1^i^	0.897 (10)	2.150 (15)	2.994 (3)	156 (3)

Symmetry code: (i) 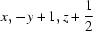.

**Fig. 1.**
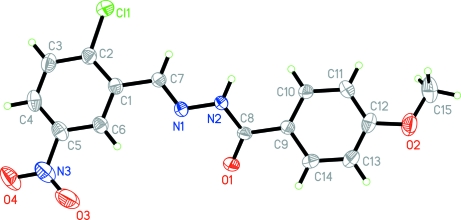
The molecular structure of the title compound, showing 30% probability displacement ellipsoids for the non-hydrogen atoms.

**Fig. 2.**
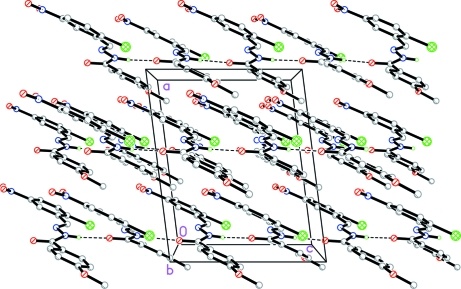
Packing diagram of the title compound, viewed along the b axis. Hydrogen bonds are shown as dashed lines. Hydrogen atoms not involved in hydrogen bonds are omitted for clarity.

## supplementary crystallographic information

### Comment

Hydrazones derived from the condensation of aldehydes with hydrazides have been demonstrated to possess excellent biological activities (Zhong *et al.*, 2007; Raj *et al.*, 2007; Jimenez-Pulido *et al.*, 2008). Due to the easy synthesis of such compounds, a great deal of hydrazones have been synthesized and structurally characterized (Yehye *et al.*, 2008; Fun, Patil, Jebas *et al.*, 2008; Yang *et al.*, 2008; Ejsmont *et al.*, 2008). We report herein the crytal structure of the title new hydrazone compound.

In the structure of the title compound (Fig. 1), the molecule exists in a *trans* configuration with respect to the methylidene unit. The dihedral angle between the two substituted benzene rings is 0.4 (3)°. In the 2-chloro-5-nitrophenyl unit, the nitro group is slightly twisted from the mean plane of the C1–C6 ring with a dihedral angle of 7.4 (3)°. The same pattern can ben observed in a similar hydrazone compound (Fun, Patil, Rao *et al.*, 2008). In the 4-methoxyphenyl unit, the methoxy group is nearly coplanar with the mean plane of the C9–C14 ring, with atom C15 deviating from the C9–C14 ring by 0.098 (2) Å. The C7-N1-N2-C8 torsion angle is 7.3 (3)°. The bond distances and angles are in normal ranges (Allen *et al.*, 1987).

In the crystal structure, molecules are linked by intermolecular N—H···O hydrogen bonds (Table 1), to form chains parallel to the *c* axis (Fig. 2).

### Experimental

The compound was prepared by refluxing 2-chloro-5-nitrobenzaldehyde (1.0 mol) with 4-methoxybenzohydrazide (1.0 mol) in methanol (100 ml). Excess methanol was removed from the mixture by distillation. The colourless solid product was filtered, and washed three times with methanol. Colourless block crystals of the title compound were obtained from a methanol solution by slow evaporation in air.

### Refinement

Atom H2 was located in a difference Fourier map and refined isotropically, with the N–H distance restrained to 0.90 (1) Å. Other H atoms were placed in calculated positions (C—H = 0.93-0.96 Å) and refined as riding with U_iso_(H) = 1.2 U_eq_(C) or 1.5U_eq_(C) for methyl H atoms. A rotating group model was used for the methyl group.

### Crystal data

**Table:**

C_15_H_12_ClN_3_O_4_	*F*(000) = 688
*M**_r_* = 333.73	*D*_x_ = 1.500 Mg m^−^^3^
Monoclinic, *C**c*	Mo *K*α radiation, λ = 0.71073 Å
Hall symbol: C -2yc	Cell parameters from 1899 reflections
*a* = 11.724 (2) Å	θ = 2.7–26.0°
*b* = 13.482 (3) Å	µ = 0.28 mm^−^^1^
*c* = 9.4259 (19) Å	*T* = 298 K
β = 97.199 (3)°	Block, colourless
*V* = 1478.1 (5) Å^3^	0.20 × 0.20 × 0.17 mm
*Z* = 4	

### Data collection

**Table:**

Bruker SMART CCD area-detector diffractometer	2735 independent reflections
Radiation source: fine-focus sealed tube	2320 reflections with *I* > 2σ(*I*)
graphite	*R*_int_ = 0.015
ω scans	θ_max_ = 27.5°, θ_min_ = 2.3°
Absorption correction: multi-scan (SADABS; Sheldrick, 1996)	*h* = −12→15
*T*_min_ = 0.946, *T*_max_ = 0.953	*k* = −17→14
4284 measured reflections	*l* = −12→11

### Refinement

**Table:**

Refinement on *F*^2^	Secondary atom site location: difference Fourier map
Least-squares matrix: full	Hydrogen site location: inferred from neighbouring sites
*R*[*F*^2^ > 2σ(*F*^2^)] = 0.035	H atoms treated by a mixture of independent and constrained refinement
*wR*(*F*^2^) = 0.090	*w* = 1/[σ^2^(*F*_o_^2^) + (0.0432*P*)^2^ + 0.2836*P*] where *P* = (*F*_o_^2^ + 2*F*_c_^2^)/3
*S* = 1.04	(Δ/σ)_max_ < 0.001
2735 reflections	Δρ_max_ = 0.14 e Å^−^^3^
212 parameters	Δρ_min_ = −0.19 e Å^−^^3^
3 restraints	Absolute structure: Flack (1983), 1079 Friedel pairs
Primary atom site location: structure-invariant direct methods	Flack parameter: −0.01 (7)

### Special details

**Table:**

Geometry. All esds (except the esd in the dihedral angle between two l.s. planes) are estimated using the full covariance matrix. The cell esds are taken into account individually in the estimation of esds in distances, angles and torsion angles; correlations between esds in cell parameters are only used when they are defined by crystal symmetry. An approximate (isotropic) treatment of cell esds is used for estimating esds involving l.s. planes.
Refinement. Refinement of F^2^ against ALL reflections. The weighted R-factor wR and goodness of fit S are based on F^2^, conventional R-factors R are based on F, with F set to zero for negative F^2^. The threshold expression of F^2^ > 2sigma(F^2^) is used only for calculating R-factors(gt) etc. and is not relevant to the choice of reflections for refinement. R-factors based on F^2^ are statistically about twice as large as those based on F, and R- factors based on ALL data will be even larger.

### Fractional atomic coordinates and isotropic or equivalent isotropic displacement parameters (Å^2^)

**Table:**

	*x*	*y*	*z*	*U*_iso_*/*U*_eq_	
Cl1	0.12621 (9)	0.12406 (5)	0.37773 (10)	0.0861 (3)	
N1	0.13488 (17)	0.41377 (14)	0.18463 (19)	0.0474 (5)	
N2	0.09570 (18)	0.49328 (13)	0.2558 (2)	0.0477 (4)	
N3	0.3330 (2)	0.2029 (2)	−0.1503 (3)	0.0806 (8)	
O1	0.07475 (18)	0.58146 (13)	0.05053 (18)	0.0603 (5)	
O2	−0.0721 (2)	0.92744 (16)	0.4294 (3)	0.0838 (7)	
O3	0.3489 (3)	0.2876 (2)	−0.1823 (3)	0.1312 (12)	
O4	0.3571 (2)	0.1320 (2)	−0.2230 (3)	0.1006 (8)	
C1	0.1933 (2)	0.24746 (17)	0.1773 (2)	0.0466 (5)	
C2	0.1870 (2)	0.15042 (18)	0.2246 (3)	0.0521 (6)	
C3	0.2269 (2)	0.07102 (18)	0.1509 (3)	0.0625 (7)	
H3	0.2211	0.0067	0.1848	0.075*	
C4	0.2750 (2)	0.0880 (2)	0.0280 (3)	0.0626 (7)	
H4	0.3019	0.0356	−0.0227	0.075*	
C5	0.2829 (2)	0.1836 (2)	−0.0190 (3)	0.0574 (7)	
C6	0.2439 (2)	0.26332 (17)	0.0522 (3)	0.0506 (6)	
H6	0.2511	0.3273	0.0176	0.061*	
C7	0.1489 (2)	0.33260 (16)	0.2508 (2)	0.0478 (5)	
H7	0.1315	0.3267	0.3440	0.057*	
C8	0.06846 (19)	0.57703 (16)	0.1789 (2)	0.0437 (5)	
C9	0.0312 (2)	0.66442 (16)	0.2574 (2)	0.0432 (5)	
C10	−0.0184 (2)	0.66089 (17)	0.3825 (2)	0.0466 (5)	
H10	−0.0277	0.6001	0.4262	0.056*	
C11	−0.0548 (2)	0.7478 (2)	0.4443 (3)	0.0536 (6)	
H11	−0.0890	0.7449	0.5280	0.064*	
C12	−0.0394 (2)	0.83779 (18)	0.3803 (3)	0.0594 (7)	
C13	0.0120 (3)	0.8419 (2)	0.2577 (3)	0.0664 (7)	
H13	0.0243	0.9031	0.2166	0.080*	
C14	0.0454 (2)	0.75687 (18)	0.1949 (3)	0.0576 (7)	
H14	0.0778	0.7607	0.1099	0.069*	
C15	−0.1293 (3)	0.9299 (3)	0.5519 (5)	0.0924 (11)	
H15A	−0.1978	0.8904	0.5359	0.139*	
H15B	−0.1493	0.9972	0.5714	0.139*	
H15C	−0.0796	0.9039	0.6320	0.139*	
H2	0.085 (2)	0.490 (2)	0.3482 (13)	0.080*	

### Atomic displacement parameters (Å^2^)

**Table:**

	*U*^11^	*U*^22^	*U*^33^	*U*^12^	*U*^13^	*U*^23^
Cl1	0.1402 (7)	0.0565 (4)	0.0680 (4)	0.0130 (5)	0.0377 (4)	0.0108 (4)
N1	0.0625 (12)	0.0416 (10)	0.0391 (10)	0.0010 (8)	0.0102 (9)	−0.0058 (8)
N2	0.0704 (12)	0.0389 (9)	0.0353 (9)	0.0033 (8)	0.0128 (9)	−0.0020 (7)
N3	0.0738 (16)	0.094 (2)	0.0798 (18)	−0.0181 (14)	0.0314 (13)	−0.0263 (16)
O1	0.0928 (13)	0.0558 (9)	0.0343 (9)	0.0018 (9)	0.0157 (8)	0.0020 (7)
O2	0.0947 (15)	0.0526 (12)	0.1063 (18)	0.0148 (10)	0.0216 (13)	−0.0121 (11)
O3	0.187 (3)	0.098 (2)	0.131 (2)	−0.042 (2)	0.109 (2)	−0.0274 (17)
O4	0.1071 (17)	0.1107 (19)	0.0928 (16)	−0.0112 (14)	0.0478 (14)	−0.0467 (14)
C1	0.0483 (12)	0.0468 (12)	0.0440 (13)	−0.0013 (10)	0.0028 (10)	−0.0074 (10)
C2	0.0607 (15)	0.0487 (12)	0.0455 (13)	0.0046 (11)	0.0015 (11)	−0.0052 (10)
C3	0.0749 (17)	0.0441 (13)	0.0663 (18)	0.0083 (12)	0.0008 (14)	−0.0078 (12)
C4	0.0590 (15)	0.0581 (15)	0.0696 (18)	0.0076 (12)	0.0029 (13)	−0.0235 (13)
C5	0.0464 (14)	0.0688 (17)	0.0574 (15)	−0.0061 (11)	0.0080 (12)	−0.0217 (13)
C6	0.0500 (13)	0.0504 (13)	0.0517 (14)	−0.0037 (12)	0.0071 (11)	−0.0082 (11)
C7	0.0631 (14)	0.0425 (12)	0.0379 (12)	−0.0009 (11)	0.0067 (10)	−0.0031 (9)
C8	0.0553 (13)	0.0426 (11)	0.0339 (11)	−0.0066 (10)	0.0085 (9)	0.0000 (9)
C9	0.0510 (13)	0.0428 (11)	0.0352 (11)	0.0004 (10)	0.0028 (10)	−0.0001 (9)
C10	0.0563 (14)	0.0431 (12)	0.0399 (12)	−0.0016 (10)	0.0046 (11)	0.0018 (10)
C11	0.0512 (13)	0.0615 (16)	0.0481 (15)	0.0030 (12)	0.0064 (12)	−0.0111 (11)
C12	0.0589 (15)	0.0406 (13)	0.0752 (19)	0.0073 (11)	−0.0051 (14)	−0.0043 (12)
C13	0.0841 (19)	0.0430 (13)	0.0721 (18)	0.0036 (13)	0.0099 (15)	0.0094 (13)
C14	0.0753 (17)	0.0485 (14)	0.0507 (14)	−0.0021 (12)	0.0144 (13)	0.0100 (11)
C15	0.077 (2)	0.081 (2)	0.121 (3)	0.0114 (16)	0.017 (2)	−0.038 (2)

### Geometric parameters (Å, °)

**Table:**

Cl1—C2	1.725 (3)	C4—H4	0.9300
N1—C7	1.260 (3)	C5—C6	1.376 (3)
N1—N2	1.374 (2)	C6—H6	0.9300
N2—C8	1.358 (3)	C7—H7	0.9300
N2—H2	0.897 (10)	C8—C9	1.485 (3)
N3—O3	1.202 (4)	C9—C10	1.379 (3)
N3—O4	1.229 (3)	C9—C14	1.397 (3)
N3—C5	1.458 (4)	C10—C11	1.399 (3)
O1—C8	1.223 (3)	C10—H10	0.9300
O2—C12	1.366 (3)	C11—C12	1.376 (4)
O2—C15	1.406 (4)	C11—H11	0.9300
C1—C2	1.387 (3)	C12—C13	1.370 (4)
C1—C6	1.402 (3)	C13—C14	1.370 (4)
C1—C7	1.469 (3)	C13—H13	0.9300
C2—C3	1.389 (3)	C14—H14	0.9300
C3—C4	1.370 (4)	C15—H15A	0.9600
C3—H3	0.9300	C15—H15B	0.9600
C4—C5	1.369 (4)	C15—H15C	0.9600
			
C7—N1—N2	117.83 (18)	C1—C7—H7	120.8
C8—N2—N1	117.34 (17)	O1—C8—N2	121.9 (2)
C8—N2—H2	120.9 (19)	O1—C8—C9	120.8 (2)
N1—N2—H2	121.7 (19)	N2—C8—C9	117.29 (18)
O3—N3—O4	123.0 (3)	C10—C9—C14	118.4 (2)
O3—N3—C5	118.3 (3)	C10—C9—C8	125.4 (2)
O4—N3—C5	118.7 (3)	C14—C9—C8	116.1 (2)
C12—O2—C15	118.8 (3)	C9—C10—C11	120.7 (2)
C2—C1—C6	117.3 (2)	C9—C10—H10	119.7
C2—C1—C7	123.4 (2)	C11—C10—H10	119.7
C6—C1—C7	119.3 (2)	C12—C11—C10	119.5 (2)
C1—C2—C3	122.1 (3)	C12—C11—H11	120.3
C1—C2—Cl1	120.45 (19)	C10—C11—H11	120.3
C3—C2—Cl1	117.4 (2)	O2—C12—C13	114.9 (3)
C4—C3—C2	119.6 (3)	O2—C12—C11	125.0 (3)
C4—C3—H3	120.2	C13—C12—C11	120.1 (2)
C2—C3—H3	120.2	C14—C13—C12	120.7 (2)
C5—C4—C3	118.9 (2)	C14—C13—H13	119.7
C5—C4—H4	120.6	C12—C13—H13	119.7
C3—C4—H4	120.6	C13—C14—C9	120.6 (2)
C4—C5—C6	122.5 (3)	C13—C14—H14	119.7
C4—C5—N3	119.5 (2)	C9—C14—H14	119.7
C6—C5—N3	118.0 (3)	O2—C15—H15A	109.5
C5—C6—C1	119.6 (2)	O2—C15—H15B	109.5
C5—C6—H6	120.2	H15A—C15—H15B	109.5
C1—C6—H6	120.2	O2—C15—H15C	109.5
N1—C7—C1	118.5 (2)	H15A—C15—H15C	109.5
N1—C7—H7	120.8	H15B—C15—H15C	109.5

### Hydrogen-bond geometry (Å, °)

**Table:**

*D*—H···*A*	*D*—H	H···*A*	*D*···*A*	*D*—H···*A*
N2—H2···O1^i^	0.90 (1)	2.15 (2)	2.994 (3)	156 (3)

Symmetry codes: (i) *x*, −*y*+1, *z*+1/2.
